# Coexistence of true talon cusp and double dens invaginatus in a single tooth: a rare case report and review of the literature

**DOI:** 10.1002/ccr3.1252

**Published:** 2017-10-30

**Authors:** Hnin Nu Nu Lwin, Pyae Phyo Kyaw, Sai Wai Yan Myint Thu

**Affiliations:** ^1^ Private Dental Clinic Yangon Myanmar; ^2^ Ministry of Health and Sports Naypyidaw Myanmar; ^3^ Present address: Maxillofacial Prosthetic Service Department of Prosthodontics Faculty of Dentistry Mahidol University 6 Yothi Street Ratchathewi Bangkok Thailand; ^4^ Present address: Department of Tropical Nutrition and Food Science Faculty of Tropical Medicine Mahidol University Bangkok Thailand

**Keywords:** Co‐occurrence, dens invaginatus, developmental dental anomaly, talon cusp

## Abstract

Co‐occurrence of a talon cusp and double dens invaginatus is an extremely rare developmental dental anomaly. This case report represents a talon cusp with two dens invaginatus on a maxillary right lateral incisor. Early identification is needed for prevention of potential problems on the affected or opposing tooth.

## Introduction

Talon cusp is a relatively rare developmental anomaly of tooth characterized by an extra cusp that is present on the anterior tooth extending from at least half of the distance incisocervically 1. It was first discovered by Mitchell in 1892 2. It was called talon cusp due to its similar appearance to eagle's talon. It is one of the various types of dens evaginatus, which is found at anterior teeth 3. Dens invaginatus is a relatively rare developmental variation of dental hard tissue, present as an invagination of the surface of crown or root that is surrounded by enamel 1.

There is no sex predilection for the occurrence of talon cusp although it has been found to have a higher frequency in Asians, Native Americans, the Inuit and Arab population. Prevalence of talon cusp is highest among the permanent maxillary central incisors (55%), followed by permanent maxillary central incisors (30%). It is more commonly found in permanent dentition than deciduous dentition, in which most of the cases are reported on the maxillary central incisor. However, it occurs rarely on mandibular incisor (6%) and maxillary canines (4%) 1. Dens invaginatus is found in 0.25–41% of the population and the prevalence of this defect is lower in Western countries when compared with other parts of the world. It may predominantly occur in Chinese or Malaysian population according to the previous literature. The coronal type II invagination is the most common form of invagination, and majority of these abnormalities were found in the permanent maxillary lateral incisors. Nearly 40% of the cases occurred bilaterally 3, 4. Although there were 11 previous reports of coexistence of talon cusp and dens invaginatus as revealed from the PUB MED database 5, 6, 7, 8, 9, 10, 11, 12, 13, 14, 15; double dens invaginatus in one talon cusp has not been reported till date. Therefore, the aim of this article was to report a talon cusp associated with double dens invaginatus that is located on the palatal aspect of permanent maxillary right lateral incisor and to review its literature.

## Case Presentation

**Figure 1:**
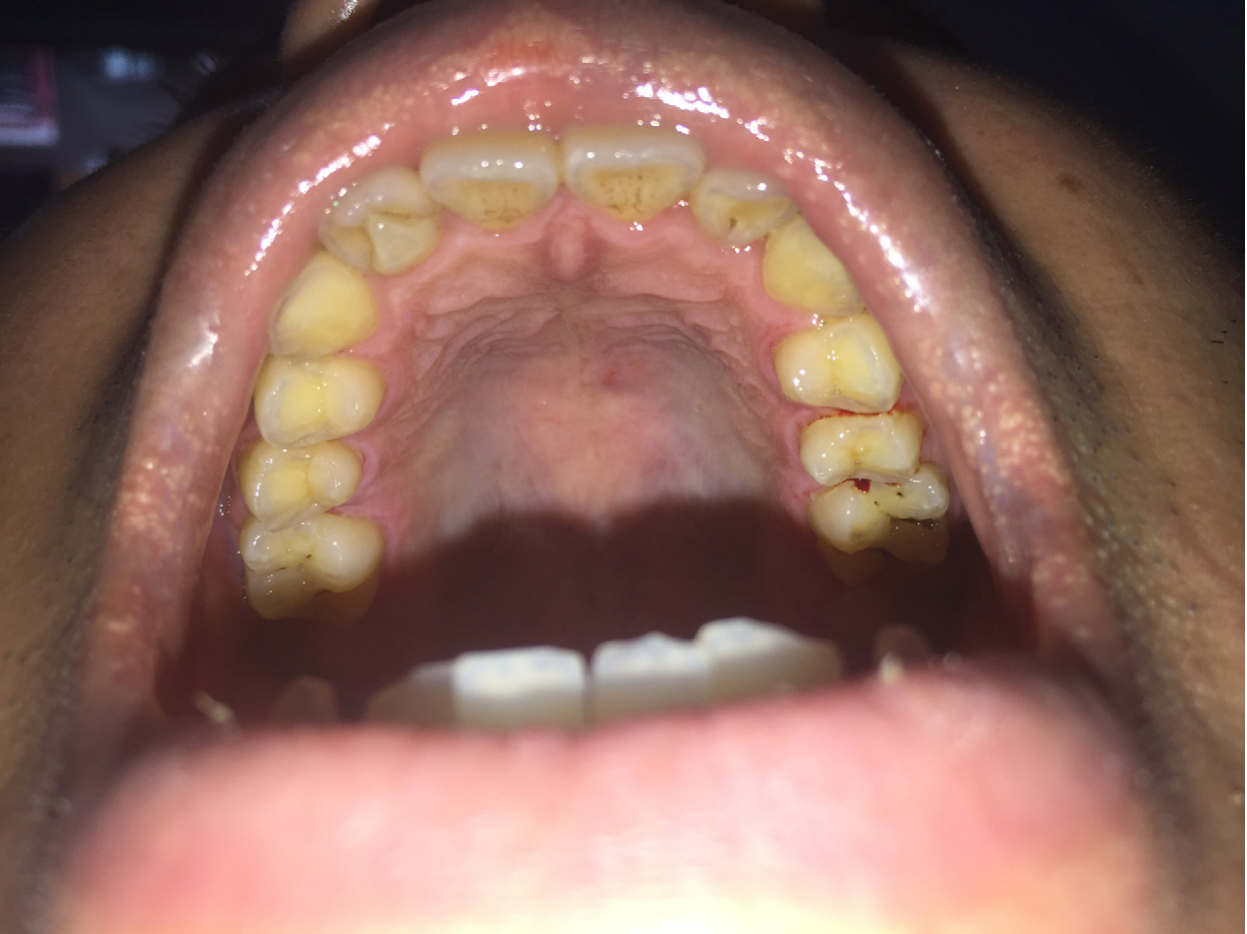
Clinical photograph of talon cusp on maxillary right lateral incisor.

**Figure 2:**
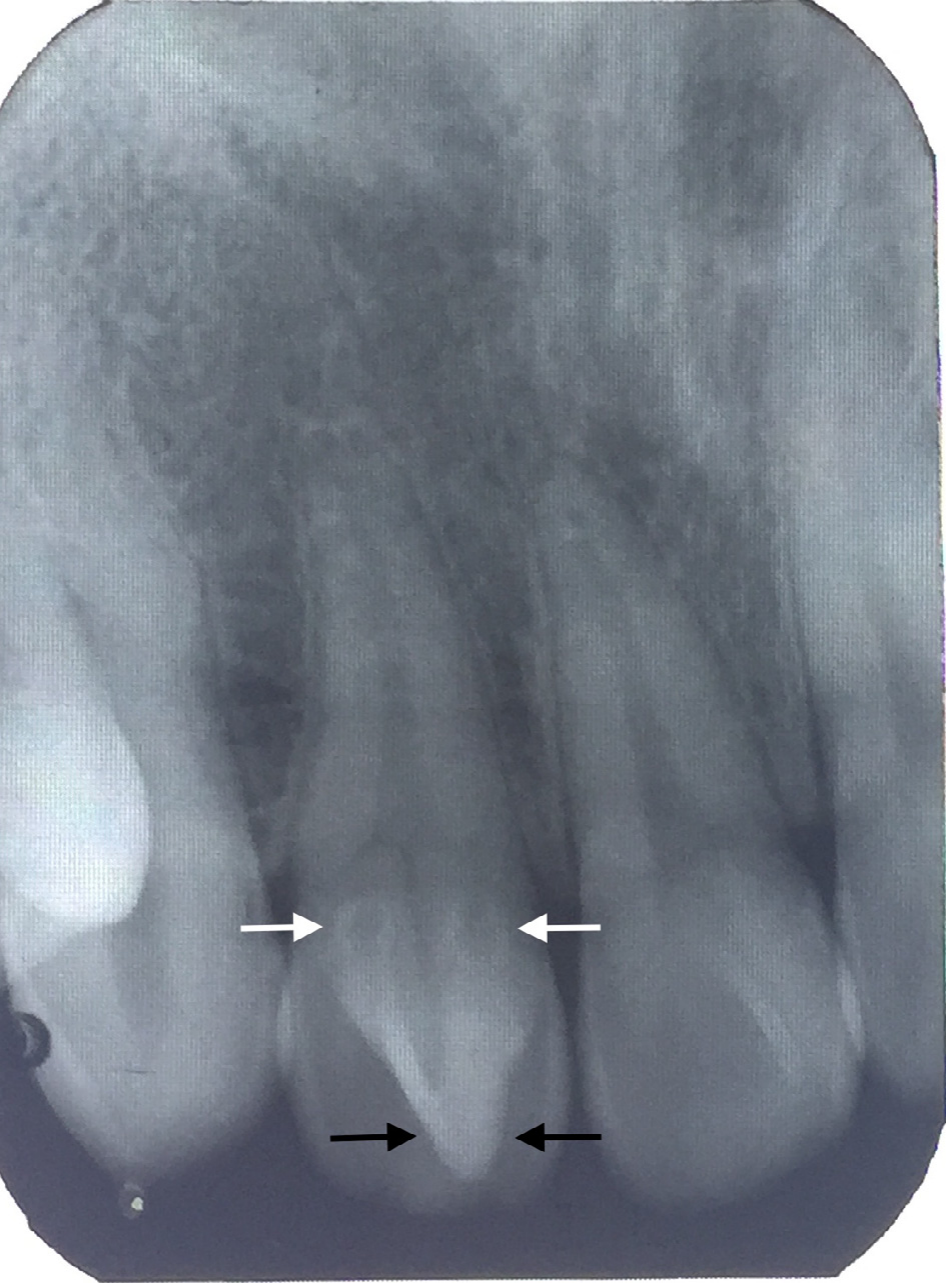
Intraoral periapical radiograph of right maxillary lateral incisor depicts talon cusp (black arrow) together with double dens invaginatus (white arrows).

**Figure 3:**
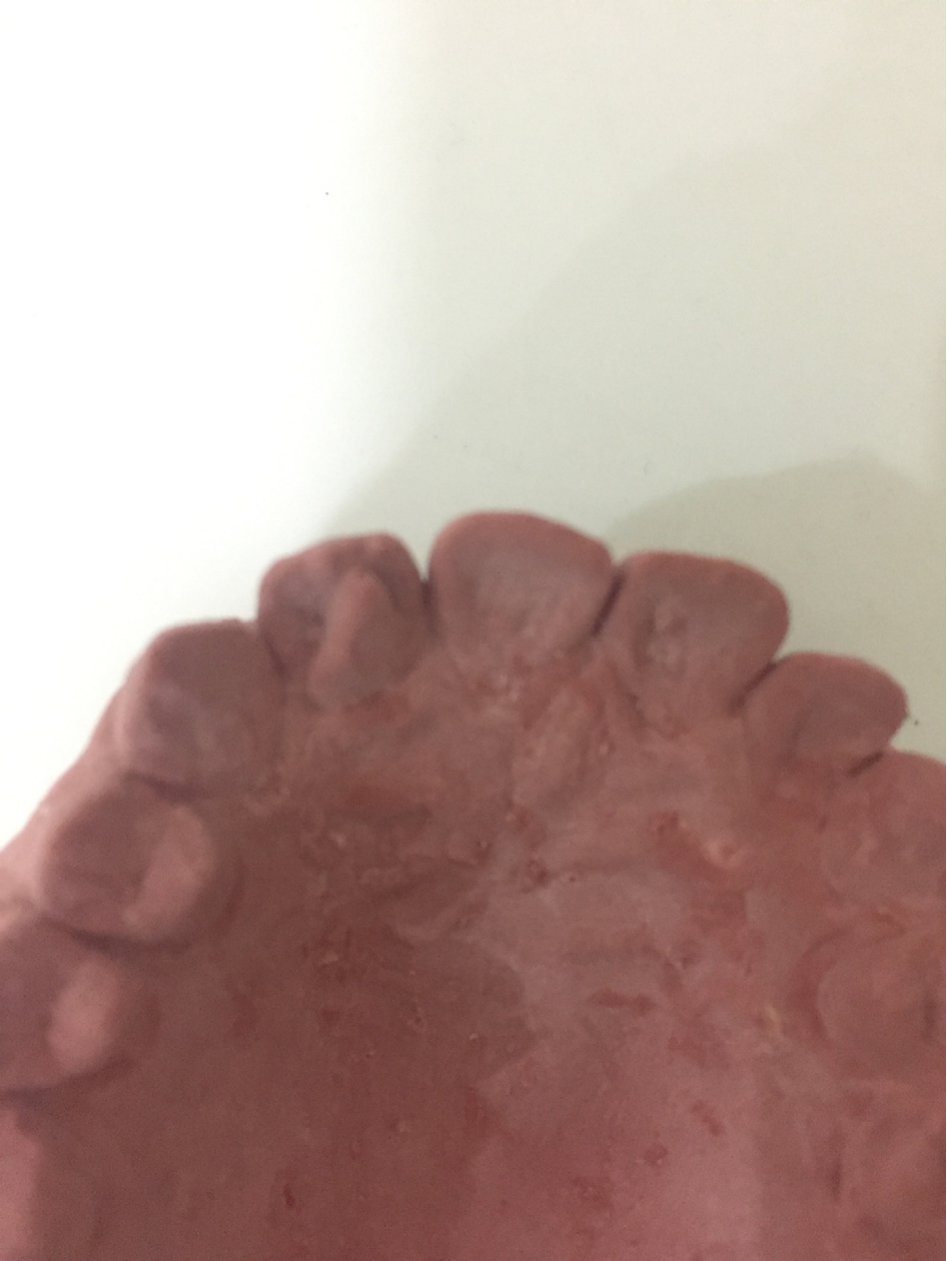
A study cast showing talon cusp on right maxillary lateral incisor.

A 30‐year‐old, healthy, male patient visited the private dental clinic for bleeding gum during tooth brushing especially at lower anterior teeth. Intraoral examination revealed two impacted mandibular last molar, fissure caries on lower first molars, calculus at upper left molar region and lower anterior teeth. He followed normal oral hygiene practice and did not receive regular dental check‐up. He did not have any systemic disease and had good general health. On clinical examination, extra cusp was detected at the palatal side of upper right lateral incisor extending from cement‐enamel junction to incisal edge, which was confirmed to be a talon cusp (Fig. 1). No caries were observed and gingiva around the affected tooth was healthy. There was no interference in occlusion as the corresponding lower tooth was located at a lingual position. Moreover, it did not cause irritation to the tongue during speech and mastication. The patient also did not suffer discomfort due to this tooth. A few months ago, he noticed that this tooth was slightly larger than normal and unsightly. However, he did not consult with any dentist at that time due to lack of pain and discomfort. His family members do not have this dental anomaly. The tooth responded well during pulp vitality test including cold test and electric pulp tester. There was no pain on percussion on this tooth both horizontally and vertically. Intraoral periapical radiograph of upper right lateral incisor (Fig. 2) showed the V‐shaped radiopaque structure consisting of enamel, dentine and a small radiolucent area of pulp tissue [black arrow]. Moreover, two keyhole‐shaped radiolucencies that are surrounded by radiopaque structures in the coronal portion of the affected tooth [white arrow]. According to the clinical and radiographic findings, the case was confirmed to be the type I true talon cusp together with double type I dens invaginatus in a permanent maxillary right lateral incisor. The study model indicated the cusp to be the size of about 4 mm wide mesiodistally and 7 mm height cervicoincisally (Fig. 3).

**Figure 4:**
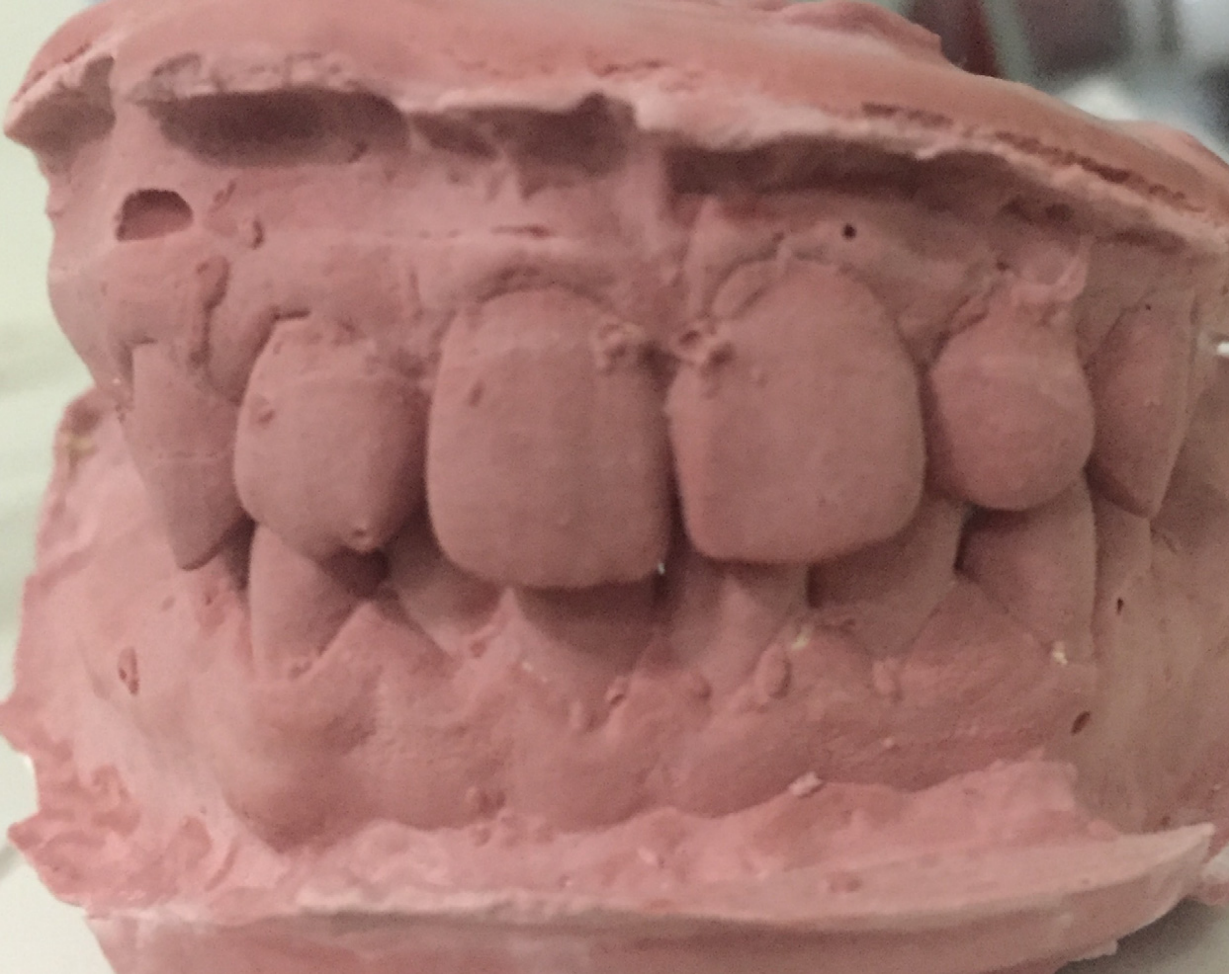
A study cast showing lingually erupted mandibular right lateral incisor.

As it enhances the plaque accumulation and development of dental caries, the developmental groove was filled with fissure sealant for preventive purpose. Moreover, there were no pathological signs and symptoms due to dens invaginatus and no additional treatment was considered. Additionally, calculus was removed from the upper left molar region and lower anterior region as the presence of talon cusp result in the eruption of corresponding lower tooth in lingual position with subsequent difficulties in accessibility of toothbrush and accumulation of plaque and calculus on the opposing tooth (Fig. 4). As the patient was not cooperated to receive orthodontic treatment of the lower anterior teeth, he was instructed to carry out proper tooth brushing practice at the lower anterior region and advised to seek regular dental check‐up. A written consent was obtained for publication of the clinical photograph, X‐ray and patient record.

## Discussion

The etiology of talon cusp is still unknown. Lots of work have been completed on human talon cusps, but a recent article has shown that baboons may also possess cases, as well 16. Some researchers reported the occurrence of this case in the same family members indicating the genetic influences of this anomaly 3, 12. It is believed that it might be a result of the aberrant proliferation of dental lamina, abnormal folding of inner enamel epithelium, focal hyperplasia of dental papilla. It was found to occur in relation with other developmental dental abnormalities such as mesiodens, odontome, unerupted or impacted teeth, peg‐shaped maxillary central incisors, dens invaginatus, fusion, bilateral germination, cleft lip and enamel clefts 17. One previous study reported that it was found to be associated with Rubinstein–Taybi syndrome, which is characterized by short stature, congenital abnormalities of craniofacial structures and mental retardation 18. This anomaly has been classified into three types based upon differences in size and shape 19.

Type 1: Talon –It is a distinctive form of extra cusp on palatal surfaces of primary or permanent anterior tooth extending from at least half of the distance incisocervically.

Type 2: Semitalon –It refers to an additional cusp that is present in the anterior tooth that only has a size of a millimeter to less than half of the distance from the cement‐enamel junction to incisal edge. It may locate at the palatal surface or strand away from the crown.

Type 3: Trace talon –It represents an enlarged form of cingula, which may present as different shape such as conical, tubercle or bifid 20.

Dens invaginatus is caused by the inward folding of the dental hard tissues before their mineralization. It might be due to focal growth retardation of part of the enamel organ while remaining part of the enamel organ keeps growing. Coexistance of dens invaginatus and other developmental dental defects such as double teeth, shovel‐shaped incisor, multi‐rooted teeth and talon cusp were reported in the previous case reports 3. Coronal invagination can be classified into three types depending on the extent of folding of dental hard tissue. Type I invagination represents folding of tooth surface that is limited to the crown of the tooth, whereas type II invagination goes beyond the cement‐enamel junction. On the other hand, type III passes over the root and ends in the apical or lateral radicaular area without any direct relationship with the pulp 1.

Although talon cusp is uncommon developmental anomaly of dental hard tissue, large‐sized talons cusps contributing to esthetic problems, occlusal interference, irritation of tongue during its functional movement, increase risk of caries in developmental groove of the cusp, increase risk of attrition and gingival periodontal disease due to extreme occlusal forces on corresponding tooth in the opposite dental arch 17, 21. Owing to the fact that dens invaginatus is characterized by extension of lingual pits and hence it contributes to the area of plaque accumulation and subsequent increase in the risk of early tooth decay, pulp and periapical inflammation 4. Although a large talon cusp often causes occlusal interference, the current case did not cause any disturbance in occlusion due to lingually erupted lower counterpart. However, it contributed to malalignment of the lower anterior tooth that enhances the plaque accumulation followed by progression into periodontal problem. The tooth did not have any carious lesions and pulpal involvement in the area of lingual pits.

Based on the previous research findings, both talon cusp and dens invaginatus were most commonly found in maxillary lateral incisors unilaterally 1, 22, 23, which supported the findings of this study. In spite of being found in the most common location of talons cusp and dens invaginatus, in this case, there has been no case report of these anomalies in the Myanmar population based on author's knowledge. There were no local problems related with co‐occurrence of dens invaginatus and talon cusp that were found in the most of the previous case reports 5, 7, 8, 12.

Management of talon cusp depends on clinical presentations and complications of the individual case. Treatment is usually not required if the size is small or it does not cause any clinical problems 21. If it has a deep developmental groove, fissure sealant should be applied in order to prevent caries in the affected tooth. In the case of occulsal interference, selective grinding of the affected tooth should be carried out gradually and regularly in combination with topical fluoride application for the prevention of hypersensitivity and enhancement of secondary dentine formation. In some cases, the total reduction in the cusp is necessary together with the use of calcium hydroxide pulpotomy. Orthodontic treatment should be considered in condition when there is an incorrect alignment of the affected tooth or corresponding tooth 17. Preventive fissure sealant is usually enough for the tooth with type I dens invaginatus, which does not show any pulpal and periapical pathology. Conventional endodontic treatment and apexification should be considered in case of type II invagination and surgical endodontic therapy should be carried out for type III invagination 3. Talon cusp and den invaginatus of this case did not cause any significant problems to the patient. Nevertheless, it caused problems for the opposing lower counterpart as it predisposed to the eruption of corresponding lower tooth in a lingual position with the subsequent predisposition to plaque accumulation and gum disease.

## Conclusion

Talon cusp and dens invaginatus are the developmental abnormalities, which are not harmless. As it may lead to many hurdles during diagnosis and treatment planning of the affected tooth for the dentists. Therefore, regular follow‐up of these developmental dental defects and careful brushing of corresponding lower teeth is critical for prevention of negative effects. Furthermore, early identification and immediate management are needed to give a necessary orthodontic treatment of the corresponding tooth in order to prevent complications such as plaque accumulation and gingivitis.

## Conflict of Interest

No conflict of interest related to this article is reported.

## Authorship

HNNL: contributed to conception or design of the work, acquisition of data, completed the literature review, wrote the first draft of the manuscript, obtained consent from the patient, revised for critically important intellectual content and involved in final approval of the manuscript for publication. PPK: contributed to conception or design of the work, reviewed the literatures, wrote the first draft of the manuscript, revised it critically for important intellectual content and involved in final approval of the manuscript for publication. SWYMT: contributed to conception or design of the work analysis and interpretation of data, verified literature review, wrote the first draft of the manuscript, revised it critically for important intellectual content and involved in final approval of the manuscript for publication.
